# The Role of Radiation, Immunotherapy, and Chemotherapy in the Management of Locally Advanced or Metastatic Cutaneous Malignancies

**DOI:** 10.3390/cancers16233920

**Published:** 2024-11-22

**Authors:** Irini Yacoub, Kareem Rayn, J. Isabelle Choi, Richard Bakst, Arpit Chhabra, Joshua Y. Qian, Peter Johnstone, Charles B. Simone

**Affiliations:** 1 New York Proton Center, New York, NY 10035, USA; 2 Department of Radiation Oncology, Moffitt Cancer Center, Tampa, FL 33612, USA; 3 Department of Radiation Oncology, Memorial Sloan Kettering Cancer Center, New York, NY 10065, USA; 4 Department of Radiation Oncology, Mount Sinai Medical Center, New York, NY 10029, USA

**Keywords:** cutaneous, basal cell, squamous cell, Merkel cell, melanoma, immunotherapy, radiotherapy, systemic therapy

## Abstract

Introduction: Skin cancer impacts a significant proportion of the population. While surgical management is often the mainstay of treatment, advanced or metastatic cutaneous malignancies require additional local and/or systemic therapies. Methods: A review of the literature was performed studying the use of radiation therapy, chemotherapy, and immunotherapy for locally advanced or metastatic cutaneous malignancies. Results: A summary of the present literature on the management of locally advanced or metastatic cutaneous malignancies is presented across cutaneous head and neck basal cell carcinoma, squamous cell carcinoma, melanoma, and Merkel cell carcinoma. The addition of multidisciplinary therapies to resection is often associated with improved outcomes. Conclusion: The management of cutaneous head and neck malignancies requires an approach integrating multiple specialties, to optimize outcomes and minimize toxicities.

## Simple Summary

Skin cancer impacts many people, and while surgery is often the primary treatment, more advanced cases require additional therapies. We reviewed the literature on radiation therapy, chemotherapy, and immunotherapy for advanced or metastatic skin cancers. Our findings show that combining these therapies with surgery can lead to better outcomes for cutaneous malignancies. Thus, a multidisciplinary approach that includes surgery and various adjunct treatments is crucial for effective management of these conditions.

## 1. Introduction

The skin comprises three layers, the epidermis, dermis, and hypodermis. Cells located within the epidermis include keratinocytes, melanocytes, and Langerhans and Merkel cells [1]. Keratinocytes are the dominant cell types of the skin and play a role in skin repair, barrier protection, and immunity [1]. Keratinocyte carcinomas comprise basal cell and squamous cell carcinomas [2]. Melanocytes produce melanin, responsible not only for skin color but also for protection of the skin from UV radiation [3]. These cells are involved in the etiology of melanoma. Merkel cells are touch-sensitive cells important for neural encoding of light touch stimuli [4], and can transform to Merkel Cell Carcinomas.

Cutaneous malignancies are the most commonly diagnosed malignancies in the United States and worldwide. Although these cancers are often adequately treated by resection alone, advanced stage disease may be locally infiltrative, has a more guarded prognosis, and can cause high morbidity, warranting the use of multimodal therapy. We review the use of radiation therapy (RT), systemic therapy, and immunotherapy in the management of cutaneous head and neck malignancies.

## 2. Materials and Methods

A thorough review of the literature for the best available evidence on the management of locally advanced and metastatic cutaneous malignancies was performed. Pubmed was searched using keywords including “cutaneous” “radiation”, “immunotherapy”, “systemic therapy”, “merkel cell”, “melanoma” “basal cell”, and “squamous cell”.

## 3. Results

### 3.1. Basal Cell Carcinoma

#### 3.1.1. Introduction

Basal cell carcinoma (BCC) is the most common cutaneous malignancy, making up about 70–80% of all cutaneous cancers [5]. Ultraviolet exposure is a significant risk factor for the development of BCC [6]. BCC is not typically prone to lymphatic or distant metastases, but can be locally destructive [7]. The rate of metastases has been estimated to be between 0.003% and 0.5% [8].

Cutaneous basal cell carcinoma of the head and neck is commonly managed by surgical resection alone [9]. However, radiation therapy may be used for BCC in the adjuvant setting for locally advanced stages, or when surgery is excluded due to cosmetic or functional reasons [10]. In the adjuvant setting, radiation therapy is typically recommended for high-risk factors including, but not limited to, close or positive margins, perineural invasion, recurrent disease, nodal metastases, certain types of or poorly differentiated histology, and bone invasion [11].

#### 3.1.2. Radiation for Positive Margins

Positive margin rates as high as 24% have been reported in the literature, with tumor locations in the face comprising a majority of these cases [12]. Liu et al. conducted a large study of patients treated at the Princess Margaret Cancer Center, comparing observation to irradiation for patients with incompletely resected BCC. They reported on 187 patients included in the analysis, of whom 109 of whom were irradiated, 67 were managed expectantly, and 1 was re-resected. Median radiation dose was 3500 cGy (range 1000–5000 cGy) in five fractions using orthovoltage radiation therapy. Patients treated immediately with adjuvant RT had 5- and 10-year relapse-free rates of 91% and 91%, whereas those observed had inferior relapse rates of 61% and 40%, respectively (*p* = 0.0001) [13].

#### 3.1.3. Radiation Therapy for High-Risk Factors

BCC with perineural invasion (PNI) is associated with higher recurrence and poorer prognosis [14]. The extent of perineural invasion has been shown to be prognostic. In an evaluation of 35 patients with perineural spread of SCC and BCC, the 5-year overall survival (OS) for patients with radiographic evidence of PNI was 50%, compared to 86% for patients with imaging negative PNI [15]. A study by Lin et al. analyzed the outcomes of 56 patients with SCC or BCC (12 with BCC) with perineural invasion. Forty-one patients received surgery and RT, whereas 15 received RT alone. Only three patients in the BCC group had suffered a recurrence, with a median time to recurrence of 33 months and a 5-year RFS of 80% for the BCC group [16]. These data are limited by small numbers, however, and very few data are available, if any, comparing the benefit of adjuvant RT to resection alone in patients with PNI.

#### 3.1.4. Radiation Therapy in the Definitive Setting

Definitive RT is also typically recommended as a curative treatment modality in patients with BCC who are unable to undergo surgical resection due to advanced disease [17]. Al-Othman et al. evaluated outcomes in patients with skin cancers of the head and neck who had T4 stage disease, defined as those with tumors that invade deep extradermal structures such as cartilage, muscle, bone, or nerve. The authors demonstrated ultimate local control (LC) rates of 90% at 5 years and 85% at 10 years [18]. These findings demonstrate that RT is a viable option and may improve the chance of cure in patients with very advanced disease unable to undergo resection.

#### 3.1.5. Chemotherapy

The role of chemotherapy for locally advanced and metastatic BCC is unclear. Pfeiffer et al. conducted a systematic review of the literature on cytotoxic therapy for BCC. Of the 53 patients analyzed, 28 patients received non-cisplatin-based therapy, with one partial response being reported, whereas 17 out of 22 (77%) patients who received cisplatin-based therapy had some response, including 10 who had complete disappearance of disease. Survival ranged from 4 to 51 months (median 22 months) [19]. In a subsequent report from those investigators on 53 patients receiving platinum-containing chemotherapy, the overall response rate (ORR) reported was 83%, with a complete response in 37% and a median time to progression of 24 months [20]. While platinum-based therapy can effectively induce responses in advanced BCC, the duration of response varied in these reports, and without randomized trials, the survival benefit and impact on quality of life from platinum-based therapy remain uncertain.

#### 3.1.6. Targeted Therapy

**Figure 1:**
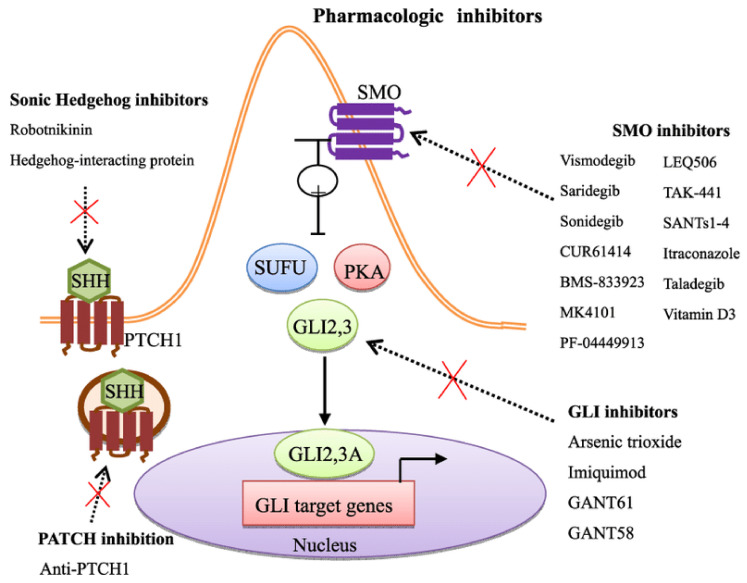
Hedgehog signaling pathway demonstrating pharmacologic inhibitors of the signaling cascade and its molecular targets [25].

Aberrant signaling in the sonic hedgehog pathway (SHH) has been implicated in the development of BCC (Figure 1). In a phase I trial of vesmodegib in 33 patients with metastatic or locally advanced BCC, 18 patients with metastatic disease had an objective response, 11 had stable disease, and 4 patients had progressive disease. In addition, 9 of 15 patients with locally advanced disease had a partial or complete response [21]. In a larger phase 2 study of vismodegib in locally advanced or metastatic BCC, the response rate for 63 patients with locally advanced disease was 60%, with 20 patients (32%) having a complete response, and median duration of response of 7.6 months, and median progression-free survival (PFS) for patients with locally advanced disease of 9.5 months [22]. Sonidegib, another HH pathway inhibitor, demonstrated long-term efficacy and safety, with a median duration of response in LA and metastatic BCC of 26 months and 24 months, as well as a 2-year OS rate of 93% and 69%, respectively [23]. Finally, the role of these targeted medications is being explored in the neoadjuvant setting, in order to reduce surgical morbidity. Vismodegib has shown favorable outcomes in this setting in patients with LA BCC, as demonstrated by the VISMONEO trial, where 27 of 44 patients had a complete response, with a treatment duration of 6 months [24].

#### 3.1.7. Immunotherapy

**Figure 2:**
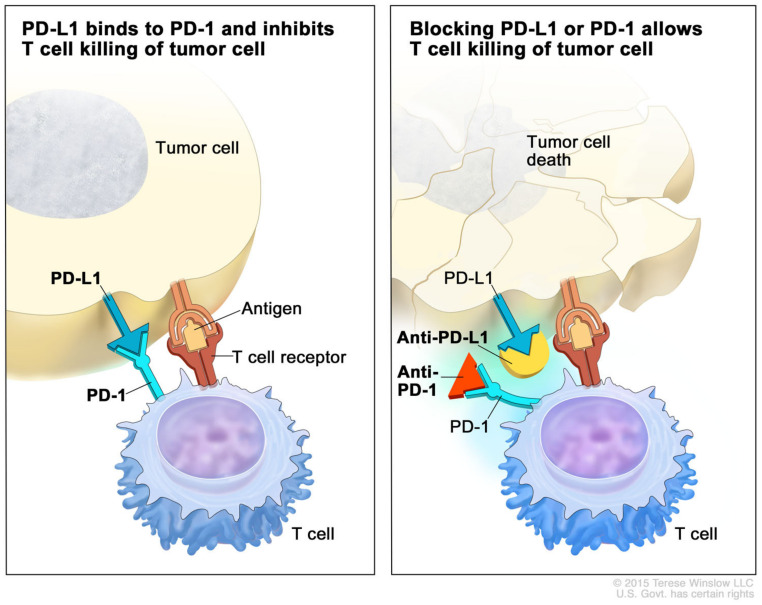
Mechanism of action of PD-1 and PD-L1 inhibitors. Tumor cells express PD-L1, which interacts with PD-1 on T cells, inhibiting the T cells’ ability to attack the tumor cells (**left**). PD-1 and PD-L1 inhibitors block this interaction, allowing T cells to effectively eliminate tumor cells (**right**). PD-1 = programmed cell death protein 1; PD-L1 = programmed cell death ligand 1. For the National Cancer Institute © 2015 Terese Winslow LLC, U.S. Govt. has certain rights.

PD-L1 expression has been shown to be as high as 90% in BCC tumor cells [26]. The binding of PD-1 on T cells to PD-L1 ligands transmits a downstream signal to the T cell to inhibit cytokine production and inhibit T cell proliferation (Figure 2). This effectively increases immune tolerance to foreign antigens, like malignant cells. PD-1 and PD-L1 inhibitors are immune checkpoint inhibitors that block this interaction and allow for an immune response to tumor cells [27]. BCCs also have a high tumor mutational burden (TMB), increasing the presence of neoantigens that are targeted by the immune system [28]. Stratigos et al. performed a phase 2 trial evaluating the efficacy of cemiplimab, a PD-1 antibody, on 84 patients with metastatic or locally advanced BCC who progressed on hedgehog therapy. Five patients had a complete response, and 21 patients had a partial response [29]. In 2021, the FDA approved cemiplimab for patients with locally advanced and metastatic BCC previously treated and progressed on hedgehog pathway inhibitors. Pembrolizumab, a monoclonal antibody that inhibits the PD-1 receptor, was also evaluated in a study of 16 participants, 9 receiving pembrolizumab alone and 7 receiving pembrolizumab and vismodegib. Of the 9 patients who received pembrolizumab alone, 44% (*n* = 4) achieved partial responses, with a median duration of response of 67.6 weeks. Among the 7 patients receiving pembrolizumab with vismodegib, 29% (*n* = 2) achieved a partial response, for a median duration of response of 52.8 weeks. The median time to response was 10.4 weeks. The one-year PFS was 70%, and OS was 94% for all subjects [30]. Finally, the role of immunotherapy is being evaluated neoadjuvantly for patients with locally advanced disease, with case series showing favorable results [31]. Trials are needed in the neoadjuvant setting to study the impact of neoadjuvant immunotherapy in LA and metastatic BCC. Overall, the findings suggest a promising role for immunotherapy in advanced or metastatic BCC.

### 3.2. Squamous Cell Carcinoma

#### 3.2.1. Introduction

Cutaneous squamous cell carcinoma (cSCC) is the second most common nonmelanoma skin cancer after BCC. The most significant risk factor for cSCC is UV radiation [32]. The prognosis of cSCC is generally excellent, although up to 5% of patients may have nodal metastases, and 1–2% of patients die of cSCC [33]. The current standard of care for the management of cSCC is surgical resection, followed by risk adjusted adjuvant treatment. The National Comprehensive Cancer Network currently classifies patients with cSCC into low-risk, high-risk, and very high-risk categories based on the risk of local recurrence, metastases, and death (Table 1). The presence of these high-risk factors after resection often necessitates the use of adjuvant therapies.

#### 3.2.2. Radiation Therapy

Radiation therapy is frequently used in the adjuvant setting for high-risk disease or locally advanced disease. The most common adjuvant indication is margin status, where RT has shown to reduce recurrence rates [17]. Perineural invasion is also associated with a poor prognosis, higher metastasis rates, and increased mortality in cSCC [34,35]. In these cases, adjuvant RT can reduce recurrence rates, especially in patients with clinically apparent PNI, which carries a worse prognosis than pathologic PNI [14,36,37,38,39].

Postoperative RT (PORT) is strongly recommended for locally infiltrative or advanced disease. The TROG 05.01 phase III trial compared postoperative chemoradiotherapy (CRT) to RT alone in 310 patients with primary disease larger than 5 cm, invasion of bone, cartilage or muscle, or nodal involvement. The study found beneficial outcomes for both groups, with locoregional control rates of 88% at 2 years and 83% at 5 years for RT alone, and 89% and 87% for CRT, respectively [40]. Adjuvant RT also improved survival in patients with regional node involvement [37]. For locally advanced, inoperable cases, RT has shown promising results. Marconi et al. reported local control rates of 92% at 5 years and 87% at 10 years [41]. Similarly, Cognetta et al. demonstrated control rates of 98.2% at 2 years and 94% at 5 years in 994 cSCC lesions [42].

#### 3.2.3. Chemotherapy

The use of chemotherapy for cutaneous SCC is generally reserved for advanced or metastatic disease. The current recommendations are based largely on retrospective studies, with very few patients studied. Initial reports using cisplatin and doxorubicin demonstrated response rates of up to 33% and partial response rates of up to 50% [43,44]. Sadek et al. treated 14 patients with cisplatin, 5-FU, and bleomycin and reported an objective response rate of 84%, including a 30% complete response rate [45]. The addition of interferon alpha (IFNalpha) and cis-retinoic acid to cisplatin demonstrated an ORR of 34%, with a median duration of 35 months [46]. These studies suggest combination therapies are likely more efficacious than single-regimen cytotoxic therapies. Additionally, in many of these studies, chemotherapy was combined with other local therapies to optimize local control. Notably, Nottage and colleagues performed a prospective study of definitive chemoradiation in 21 patients with locally or regionally advanced cSCC. Of 19 evaluable patients, 10 achieved a complete response to CRT, for a CR rate of 53%, and 2 additional patients were disease free after further resection of the disease. All 19 patients achieved a complete or partial response [47]. While these results are promising, the addition of other therapies, including RT, certainly confounds the true benefit of systemic therapy, if any. Another limitation of these regimens is their toxicity, especially when given to elderly and/or frail populations.

#### 3.2.4. Immunotherapy

Patients who are immunosuppressed tend to have more aggressive cSCC and are at increased risk of local recurrence, regional and distant metastasis, and mortality [48]. Cutaneous SCC has a high tumor mutational burden, which is often associated with a robust response to immune checkpoint inhibitors. However, in patients who are chronically immunosuppressed, there is impairment of the normal immune surveillance of the tumor environment [49].

One of the earliest trials evaluating immunotherapy in cSCC was conducted by Migden et al. in 2018. The authors studied the impact of cemiplimab in an expanded cohort of a phase I study of patients with advanced or metastatic cSCC not amenable to surgery or RT. The authors reported a 50% response rate in patients with locally advanced cSCC and 47% in patients with metastatic cSCC. The duration of responses exceeded 6 months in nearly 60% of patients, with 82% maintaining a response at the time of data cutoff [50]. Cemiplimab has also been explored in the neoadjuvant setting in patients with locally advanced, resectable cSCC and demonstrated a 70% pathologic CR rate [51]. This was further evaluated in a larger phase II study of 79 patients prior to curative intent resection and showed a 51% pCR rate and 15% major response rate [52].

Pembrolizumab has also gained interest for cSCC and demonstrated promising outcomes. Maubec et al. evaluated the outcome of first-line pembrolizumab in patients with unresectable cSCC and reported an ORR of 41% and median OS of 25 months, with patients having PD-L1+ status having a higher response (55%) compared to patients with negative PD-L1 status. Additionally, there was only a 7% grade 3 toxicity rate [53]. Similarly promising findings were demonstrated by Grob and colleagues in the phase II KEYNOTE-629 study, in which patients with recurrent and/or metastatic cSCC not amenable to surgery or RT received pembrolizumab every 3 weeks. The ORR was 35%, and disease control was 52%. The median duration of response and OS were not reached. The most common adverse events were pruritis, asthenia, and fatigue, although six patients had grade 3–5 adverse events [54]. Nivolumab, an agent with a similar mechanism to pembrolizumab, demonstrated an ORR of 58% in patients with locally advanced or metastatic cSCC, although there were no complete responses. The median duration of response was not reached, and median OS was 20.7 months [55]. For patients who are resectable, there may be a role for neoadjuvant immunotherapy to minimize cosmetic and functional deficits with surgery. In the De-Squamate study, 27 patients received neoadjuvant pembrolizumab, with 15% showing a pCR and 48% showing a cCR [56]. Overall, these studies suggest that PD-1 inhibition in patients with cSCC can achieve durable responses with generally tolerable toxicities. Overall, these studies suggest that PD-1 inhibition in patients with cSCC can achieve durable responses with generally tolerable toxicities.

### 3.3. Melanoma

#### 3.3.1. Introduction

The incidence of primary cutaneous melanoma has steadily increased over the past few decades, and it remains the deadliest form of skin cancer [57]. Sun exposure is a key driver of cutaneous melanoma development [58]. Prognosis depends on the stage at diagnosis, with 84% of patients diagnosed with localized disease, which typically has a favorable outlook, particularly for tumors 1.0 mm or thinner [59]. Survival rates drop with thicker tumors, ulceration, and high mitotic rates, with stage III 5-year survival ranging from 20% to 70%, depending on lymphatic involvement [60]. Surgical excision is the primary treatment, with studies showing that narrower margins of 1–2 cm are adequate for tumors less than 2.0 mm thick and provide no significant benefit in outcomes compared to wider margins [61].

#### 3.3.2. Radiation Therapy

Adjuvant RT plays a key role in desmoplastic neurotropic melanoma, which has a late rate of recurrence. A phase II study (NCCTG N0275, Alliance) evaluated the effectiveness of adjuvant RT in 20 adult patients with desmoplastic melanoma. No cases of regional or distant metastases were reported, and the 2- and 5-year OS rates were 95% and 77%, respectively [62]. Additionally, no serious treatment-related side effects were noted [62]. In a more recent phase III trial (NCT00975520) comparing adjuvant RT to observation after resection of neurotropic melanoma (NM) in the head and neck, local recurrence occurred in 8% of participants—three in the observation group and one in the RT group—without a difference in OS or time to relapse between the groups and no major differences in toxicities between the two [63].

Adjuvant RT has also been shown to decrease relapse in high-risk melanoma patients. A large retrospective study of 615 patients showed that regional recurrence occurred in only 10% of those who received RT, compared to 41% in non-irradiated patients, with RT associated with significantly improved locoregional control (*p* < 0.0001) [64]. The TROG 02.01 study further demonstrated reduced lymph node field relapse rates with adjuvant RT (21% vs. 36%) but found no significant difference in overall or recurrence-free survival compared to observation, with 22% of patients experiencing grade 3–4 toxicity [65].

Due to the sensitive location of these tumors, the need to reduce radiation-related toxicity is necessary. A recent study evaluated the use of proton beam therapy (PBT) for head and neck cutaneous melanoma [66]. Eight patients with predominantly advanced disease—37.5% T3 or T4 and 50% N2 or N3—with a median age of 69 years were treated with postoperative PBT after surgery. The median RT dose was 46 GyRBE, commonly delivered in 20 fractions. At a median follow-up of 40.1 months, the OS rates were 85.7% at 1 year and 35.7% at 3 years [66]. Local–regional recurrence-free survival (LRFS) was high, at 100% at 1 year and 85.7% at 3 years. Five patients experienced distant metastases, with three subsequently receiving immunotherapy. Acute toxicities of grade 2 or higher were observed in five patients, with two experiencing grade 3 toxicities, including radiation dermatitis and an immunotherapy-related rash [66]. No grade 4 or higher toxicities were reported. The study concludes that PBT provides effective local control with manageable acute toxicities, although distant metastasis remains a significant challenge [66].

#### 3.3.3. Chemotherapy

Traditional systemic therapies for melanoma, like high-dose interferon alfa (IFN alfa), have largely proven ineffective for improving long-term outcomes after surgery. While some trials showed modest improvements in relapse-free survival (RFS) and OS, other studies found no significant benefit [67,68,69,70]. A meta-analysis revealed only small gains in 5- and 10-year event-free survival and OS, with improvements of less than 4% [71].

#### 3.3.4. Targeted Therapy

Malignant transition of the melanocyte to melanoma at the molecular level depends on constitutive activation of the RAS-RAF-MEPK/ERK pathway [72]. BRAF mutations are found in more than half of patients with melanoma [73]. Therapies targeting the BRAF pathway have contributed to the improvement in survival of these patients [72]. In a trial by Chapman et al. on 675 patients with metastatic melanoma, vemurafenib demonstrated an OS rate of 84% compared with dacarbazine (64%) [74]. Unfortunately, these results are often short-lived as patients develop resistance to BRAF inhibition [75]. Studies have demonstrated combining BRAF inhibitors with inhibitors of MEK can delay the development of resistance [76,77,78,79,80]. The combination of Dabrafenib and Trametinib has also been studied in the neoadjuvant setting for resectable melanoma. Long et al. demonstrated a 49% pCR rate and a 51% pPR rate, with a median relapse free survival of 30.6 months for those with a pCR [78].

#### 3.3.5. Immunotherapy

Checkpoint inhibitors like ipilimumab, a CTLA-4 antibody, and PD-1 inhibitors have shown significant improvements in response rates, PFS, and OS in advanced melanoma (Table 2). Ipilimumab has been particularly impactful, with 20% of patients achieving long-term survival in phase III trials, showing a 5-year OS rate of 18% compared to 9% with older treatments like dacarbazine [81,82,83]. Pembrolizumab has also shown significant benefits in treating advanced melanoma, particularly in patients with unresectable stage III or IV disease. Pembrolizumab has better outcomes than chemotherapy in the Keynote-002 and Keynote-006 trials, improving PFS and OS, especially in patients with fewer prior treatments [84,85,86]. Nivolumab has also shown improved response rates, PFS, and OS in the Checkmate 037 [87], 066 [88,89], and 067 [90] trials, along with long-term survival in up to 40% of patients, and durable responses that persist even after treatment ends [89]. In the neoadjuvant setting, both CTLA-4 and PD-1 inhibitors, as monotherapy or in combination, have demonstrated extremely favorable results, with pCR rates as high as 57% [91,92,93,94,95].

### 3.4. Merkel Cell Carcinoma

#### 3.4.1. Introduction

Merkel cell carcinoma (MCC) is a rare but highly aggressive form of skin cancer, with around 2488 cases diagnosed annually in the U.S. [99]. Most cases are attributed to malignant transformation secondary to the Merkel cell polyomavirus, with a minority related to ultraviolet radiation [100]. Over 50% of MCC patients develop lymph node metastases, and over 30% develop distant metastases [101,102]. The disease has a high mortality rate, with 5-year survival rates ranging from 41% to 77%, depending on the stage at diagnosis [103,104]. Surgery is the most common and effective treatment for primary MCC [103,105].

#### 3.4.2. Radiation Therapy

The role of RT for definitive management of MCC is unclear. A study with more than 2000 patients comparing surgery with definitive RT found that surgery (with or without adjuvant RT) significantly improved OS in both stage I/II (median OS: 76 vs. 25 months; *p* < 0.001) and stage III MCC (median OS: 30 vs. 15 months; *p* < 0.001) [106]. However, the surgery group included smaller tumors, tumors in the upper extremity, and treatment at academic hospitals [106]. For patients who are poor surgical candidates or refuse surgery, initial treatment with definitive RT may still provide good outcomes. A study using SEER data showed that among non-surgical patients, those who received RT had better OS and disease-specific survival (DSS) than those who did not (5-year DSS: 73% vs. 54%; *p* < 0.0001) [107].

Several studies have consistently shown that PORT is associated with lower recurrence rates and improved survival in MCC, compared to surgery alone [3,108]. For patients with stage I/II MCC, most studies report that adjuvant RT to the primary tumor significantly reduces the rate of, and delays the time to, locoregional recurrences. Three large studies using NCDB data, with more than 1000 stage I/II patients, concluded that surgery combined with RT led to significantly better survival outcomes compared to surgery alone [109,110,111]. While adjuvant RT has shown benefits in low-risk stage I MCC, it appears that patients with high-risk features may derive the most benefit. A recent study of 1858 stage I/II MCC patients meeting high-risk criteria (e.g., positive margins, tumor size ≥ 1 cm, lymphovascular invasion [LVI]) reported a 5-year OS advantage for those who received RT when indicated (*p* < 0.003) [112].

Studies have also demonstrated the benefit of adjuvant RT in node-positive disease. A large NCDB study involving 447 SLNB-positive MCC patients showed that completion lymph node dissection (CLND) combined with adjuvant RT was associated with better OS compared to observation or RT alone [113]. Another study of MCC of the head and neck found that nodal RT in patients with positive sentinel lymph node biopsy (SLNB) significantly improved 3-year relapse-free survival (51% vs. 0%, *p* < 0.01) and regional control (95% vs. 66.7%, *p* = 0.008) [114]. Interestingly, some studies also suggest that the benefits of adjuvant RT may extend to patients with negative nodes. A retrospective analysis of 54 patients treated with surgery and RT to the regional nodes demonstrated improved regional control irrespective of nodal status (*p* = 0.01) [115]. However, the effectiveness of nodal RT in reducing nodal relapse in node-negative patients remains a topic of debate, with studies both supporting and refuting its utility [116,117].

#### 3.4.3. Chemotherapy

High-quality clinical data on adjuvant systemic therapy for MCC are limited, and chemotherapy is infrequently used in this context. Most studies show that postoperative chemotherapy, often combined with RT, does not significantly reduce recurrence or metastasis risk, nor does it improve survival [3,118,119]. In fact, two studies found that adjuvant chemotherapy was associated with worse survival outcomes [3,118]. Additionally, studies comparing postoperative chemoradiation with RT alone generally found no improvement in outcomes [120,121]. Response rates and durability of treatment are also subpar. In studies involving more than 20 patients, the ORRs typically ranged from 40% to 60%. However, the response rates depended heavily on treatment history [122,123,124]. For patients receiving first-line chemotherapy, response rates were as high as 70%, but in those who had undergone one or more previous rounds of chemotherapy, response rates plummeted to between 9% and 20% [122,123,124]. Despite some initial effectiveness, responses to chemotherapy were generally short-lived, with median durations of response ranging from 2 to 9 months. Furthermore, chemotherapy came with significant risks, particularly in older patients. The reported rates of toxic deaths ranged from 3% to 10%, making treatment decisions particularly challenging for elderly or frail patients [122,123,124]. This highlights the need for careful consideration of the risks and benefits of chemotherapy in the treatment of MCC.

#### 3.4.4. Immunotherapy

Recent clinical trials have shown promising results for the use of immune checkpoint inhibitors (ICIs) for advanced MCC [125,126,127,128,129]. Avelumab was evaluated in the JAVELIN Merkel 200 trial and demonstrated median OS of 12.6 months [125,126,127]. Additionally, the 48-month OS rate was 30%, and the 60-month OS rate was 26%. In another cohort of 116 patients who received avelumab as a first-line treatment, the ORR was 39.7%, median PFS was 4.1 months, and median OS reached 20.3 months [125,126,127]. Pembrolizumab showed similarly encouraging results in 50 patients with metastatic or locoregional MCC that could not be treated surgically or with definitive RT [128,130]. After a median follow-up of 31.8 months, the ORR was an impressive 58%, with median PFS of 16.8 months. The 3-year PFS rate was 39.1%, and the 3-year OS rate was 59.4% overall, with an even higher 89.5% OS rate in patients whose disease responded to treatment [128,130]. As seen in other cutaneous malignancies, the role of these drugs may be extended to the neoadjuvant setting. In CheckMate 358, 39 patients with stage II-IV MCC received nivolumab in the neoadjuvant setting, with 47% having a pCR, and 55% having tumor reduction ≥30% [131]. This preoperative downstaging may allow for resections with decreased morbidity.

## 4. Conclusions

The management of cutaneous head and neck malignancies requires a multidisciplinary approach, integrating surgery, radiation therapy, chemotherapy, and immunotherapy, to achieve optimal outcomes. Given the delicate location of these malignancies in the head and neck, it is crucial to harmonize these modalities to both enhance therapeutic efficacy and preserve cosmetic and functional integrity.

## Figures and Tables

**Table 1:** Risk classification for high-risk and very high-risk cutaneous SCC.

High-Risk cSCC	Very High-Risk cSCC
Tumors on trunk and extremities with diameter ≥ 2 cm and ≤4 cm	Tumors with diameter > 4 cm in any location
Tumors of any size on the head, neck, hands, feet, pretibial, and anogenital region	Poorly differentiated
Acantholytic, adenosquamous, or metaplastic subtypes, with PNI	Desmoplastic histology
PNI ≥ 0.1 mm	PNI with tumor within a nerve sheath of a nerve lying deeper than the dermis or ≥0.1 mm
Recurrent tumor	Lymphatic or vascular involvement
History of immunosuppression	>6 mm invasion
History of prior site radiation	Invasion beyond subcutaneous tissue
Poorly defined extent	
Rapid growth	
Neurologic symptoms	
2–6 mm depth	

**Table 2:** Immune checkpoint inhibitors in cutaneous melanoma: Randomized trial data for adjuvant treatment.

Trial	Phase Design	Stages Included	Treatment Arms	Median Follow-Up	RFS or DFS	DMFS	OS	AEs Any Grade; Grade 3–4; Grade 5
EORTC 18,071 [96]	III DB RCT	IIIA > 1 mm, IIIB/C no IT	HD-Ipi (*n* = 475) Pbo (*n* = 476)	5.3 y	5-y: 41% vs. 30% HR = 0.76 [0.64–0.89] *p* = 0.001	5-y: 48% vs. 39% HR = 0.76 [0.64–0.92] *p* = 0.002	5-y: 65% vs. 54% HR = 0.72 [0.58–0.88] *p* = 0.001	99% vs. 91%; 54% vs. 26%; 1.5 vs. 1.3%
CheckMate 238 [97]	III DB RCT	IIIB/C, IV	Nivo + Pbo (*n* = 453) HD-Ipi + Pbo (*n* = 453)	1.6 y	1-y: 71% vs. 61% HR = 0.65 [0.51–0.83] *p* < 0.001	1-y: 80% vs. 73% HR = 0.73 [0.55–0.95]	NR	97% vs. 99%; 25% vs. 55%, 0 vs. 0.4%
KEYNOTE-054 [98]	III DB RCT	IIIA > 1 mm, IIIB/C no IT	Pembro (*n* = 514) Pbo (*n* = 505)	1.2 y	1-y: 75% vs. 61% HR = 0.57 [0.43–0.74] *p* < 0.001	NR	NR	93% vs. 90%; 32% vs. 19%; 0.2% vs. 0

>1 mm, at least one lymph node with metastasis diameter >1 mm; AEs, adverse events; DB, double-blind; DFS, disease-free survival; DMFS, distant metastasis-free survival; HD-ipi, high-dose ipilimumab (10 mg/kg every 3 weeks for 4 doses, then every 3 months for up to 3 years); HR, hazard ratio, with 95% CI in square brackets; ipi, ipilimumab; IT, in-transit metastases; Nivo, nivolumab; NR, not reported; OL, open-label; OS, overall survival; Pbo, placebo; Pembro, pembrolizumab; RCT, randomized controlled trial; RFS, recurrence-free survival or relapse-free survival; y, year.

## Data Availability

No new data were created or analyzed in this study.
